# Elevation of high‐sensitivity cardiac troponin T at admission is associated with increased 3‐month mortality in acute ischemic stroke patients treated with thrombolysis

**DOI:** 10.1002/clc.23237

**Published:** 2019-07-23

**Authors:** Yi Sui, Ting Liu, Jianfeng Luo, Bing Xu, Liqiang Zheng, Weijin Zhao, Qi Guan, Li Ren, Chunyao Dong, Ying Xiao, Xue Qin, Yao Zhang

**Affiliations:** ^1^ Department of Neurology Shenyang First People's Hospital, Shenyang Medical College Shenyang China; ^2^ Department of Biostatistics School of Public Health, Fudan University Shanghai China; ^3^ Department of Clinical Epidemiology Shengjing Hospital of China Medical University Shenyang China; ^4^ Department of Medical Laboratory Shenyang First People's Hospital, Shenyang Medical College Shenyang China

**Keywords:** alteplase, ischemic stroke, mortality, prognosis, tissue‐type plasminogen activator, troponin

## Abstract

**Background:**

Elevated levels of cardiac troponin T (cTnT) have been associated with unfavorable outcomes in cardiac patients. However, no studies, to date, have discussed the prognostic value of high‐sensitivity cTnT (hs‐cTnT) in thrombolyzed patients with acute ischemic stroke (AIS).

**Hypothesis:**

We hypothesized that elevated levels of hs‐cTnT would be associated with poorer clinical outcomes in AIS patients treated with intravenous tissue‐type plasminogen activator (IV tPA).

**Methods:**

From January 2017 to February 2018, a total of 241 AIS patients treated with IV tPA within 4.5 hours of onset were recruited. On admission, patients were stratified into either normal or elevated hs‐cTnT groups according to a cutoff value of 14 ng/L. Multivariable logistic regression analyses were conducted to identify determinants of hs‐cTnT elevation and to detect whether elevated hs‐cTnT was associated with disability and/or mortality.

**Results:**

In multivariable regression analysis, older age (*P* < .001) and stroke etiology (*P* = .024) were significantly associated with elevated hs‐cTnT levels. After adjusting for demographic and clinical characteristics, hs‐cTnT elevation was still significantly associated with 14‐day major disability (modified Rankin Scale (mRS) 3‐5, model 1, *P =* .019, odds ratio [OR] 2.677; model 2, *P =* .015, OR 2.834), 14‐day composite unfavorable outcome (mRS 3‐6, model 1, *P =* .005, OR 3.525; model 2, *P =* .003, OR 3.976), 30‐day mortality (*P =* .049, OR 4.545) and 90‐day mortality (*P =* .049, OR 3.835).

**Conclusions:**

Elevation of hs‐cTnT at admission is associated with an increased risk of 90‐day mortality in AIS patients treated with IV tPA.

**ABBREVIATIONS**
ACS: acute coronary syndrome
AIS: acute ischemic stroke
BNP: brain natriuretic peptide
CI: confidence interval
cTn: cardiac troponin
DNT: door‐to‐needle time
ECASS: European Cooperative Acute Stroke Study
ECG: electrocardiogram
eGFR: estimated glomerular filtration rate
END: early neurological deterioration
hs‐cTn: high‐sensitivity cardiac troponin
HT: hemorrhagic transformation
IV tPA: intravenous tissue‐type plasminogen activator
MMP‐9: matrix metalloproteinase‐9
mRS: modified Rankin Scale
NCCT: non‐contrast computed tomography
NIHSS: National Institute of Health Stroke Scale
ODT: onset‐to‐door time
ONT: onset‐to‐needle time
OR: odds ratio
ROC: receiver operating characteristics
rPH: remote parenchymal hemorrhage
sICH: symptomatic intracerebral hemorrhage

## 1 INTRODUCTION

Cardiac complications, such as congestive heart failure, acute coronary syndrome (ACS), and arrhythmia occur in 20% of acute ischemic stroke (AIS) patients and are associated with unfavorable outcomes.^1^ Moreover, clear evidence indicates that cardiac lesions can be observed following nervous system insults.^2^ Increased levels of cardiac troponin (cTn), as a strong indicator of cardiac injury, have been repeatedly reported to be associated with adverse outcomes in AIS patients.^3^, ^4^, ^5^, ^6^, ^7^ Cardiac troponins exist as structural proteins in a small free pool in cardiomyocytes, and will be released in a complex form of subunits after disease conditions.^8^ Although precise etiology of elevated cTn in the setting of AIS remained to be well understood, possible causes have been reported and proposed. Catecholamine surge secondary to a sympathetic disturbance in AIS may cause cTn leakage and subsequent cardiomyocyte dysfunction.^9^, ^10^ Infarction in right insular cortex is associated with myocardial injury indicated by the elevation of cTnT.^11^ In some instances, preexisting myocardial infarction associated with cTn elevation may also complicate unfavorable outcomes of AIS.^12^ As a result, the American Heart Association/American Stroke Association (AHA/ASA) guidelines suggest a Level I recommendation of cTn assessment in all AIS patients.^13^ Furthermore, the measurement of high‐sensitivity cTn (hs‐cTn) has been increasingly utilized. Accordingly, its diagnostic value as a rapid rule‐out marker for acute myocardial infarction has been extensively investigated.^14^, ^15^, ^16^ Conversely, scarce guidelines or recommendations exist for the interpretation of cTn elevation in settings other than cardiac disease.^17^, ^18^ Therefore, the clinical implications of hs‐cTn require further elucidation. For the first time, this study examined the prognostic value of hs‐cTnT levels upon hospital admission in AIS patients treated with intravenous tissue‐type plasminogen activator (IV tPA). Additionally, the determinants of hs‐cTnT elevation in AIS patients were assessed. Finally, a cutoff value of hs‐cTnT to predict unfavorable outcomes after IV tPA was calculated by a prediction model.

## 2 METHODS

### 2.1 Study population

Consecutive AIS patients who underwent IV tPA at the Departments of Emergency Medicine or Neurology at Shenyang Brain Hospital, Shenyang Medical College, were prospectively recruited from January 2017 to February 2018. Patients were eligible for enrolment if they were diagnosed with AIS on admission and treated with IV tPA within 4.5 hours after onset. Patients were excluded from the study if increased levels of hs‐cTnT at admission were primarily attributed to an acute cardiovascular event, verified by two cardiologists. Patients younger than 18 years or beyond 4.5 hours were also excluded (Figure S1).

Clinical profiles of patients, such as demographics and previous medical history, were obtained upon admission. Clinical durations, such as onset‐to‐door time, door‐to‐needle time, and onset‐to‐needle time, were recorded by stroke nurses. Previous medical histories included smoking, alcohol consumption, hypertension, hyperlipidemia, diabetes mellitus, coronary artery disease (CAD), myocardial infarction, atrial fibrillation (AF), congestive heart failure (HF), stroke, chronic obstructive pulmonary disease, and epilepsy.

Stroke severity was assessed by certified raters using the National Institutes of Health Stroke Scale (NIHSS) and the modified Rankin Scale (mRS) scores. Stroke etiology was determined with the Trial of ORG 10172 in the Acute Stroke Treatment (TOAST) classification.^19^ Early neurological deterioration (END) was defined as a four‐point or greater NIHSS increase within 24 hours of tPA treatment.^20^

In this study, we defined symptomatic intracerebral hemorrhage (sICH) as per European Cooperative Acute Stroke Study II (ECASS II) criteria.^21^ Definitions and determination of intracranial bleeding were summarized in Appendix S1. We also defined major disability as an mRS of 3 to 5 points and composite unfavorable outcome as an mRS of 3 to 6 points at 14, 30, and 90 days after treatment.

This study was approved by the ethical review committee of Shenyang First People's Hospital, Shenyang Brain Institute, Shenyang Medical College Affiliated Shenyang Brain Hospital. All patients were informed that data may be used for research purposes, and their rights to decline participation. Written consents were not collected because of the nature of the observational study and being routine clinical practice suggested by AHA/ASA guidelines. The waiver of written consent was approved by the ethical committee. All patient‐related information was de‐identificated when subject to statistical analysis.

Levels of serum hs‐cTnT ≤14 ng/L were defined as a Reference 22, and levels of serum hs‐cTnT were considered abnormal if measured as >14 ng/L.

Characteristics of study conduction, examination of CT scan, laboratory tests, and IV tPA protocol were summarized in Appendix S1.

### 2.2 Statistical analysis

Quantitative variables were expressed as the mean and SD or median and interquartile range (IQR), and categorical variables were expressed as frequencies (%). The Student *t* test, Pearson *χ*² test, and Cochran Mantel Haenszel *χ*² were used to compare the differences between the elevated hs‐cTnT and normal hs‐cTnT groups. Univariate and multivariate logistic regression models were used to explore the influence factors of elevated hs‐cTnT and the association between elevated hs‐cTnT and functional outcomes. We used two multivariate regression models to analyze unfavorable outcomes. In model 1, we adjusted for age, gender, estimated glomerular filtration rate (eGFR), insular cortex involvement, and NIHSS on admission. In model 2, we adjusted for CAD, AF, HF, and those included in model 1. Because death was a low‐incidence event in this study, we adjusted for only age and admission NIHSS for this endpoint. To assess the discriminative capacity of serum hs‐cTnT for mortality at 30 and 90 days, the area under the receiver operating characteristics (ROC) curves were calculated. Survival analysis were estimated by the Kaplan‐Meier method and compared by the log‐rank test. All statistical inferences were two‐sided and *P* < .05 was defined as significant. STATA version 15.0 and SAS 9.3 were used for all statistical calculations.

## 3 RESULTS

### 3.1 Patient characteristics

A total of 349 patients were consecutively admitted. Patients with onset‐to‐door time exceeding 4.5 hours (n = 68), failure to receive thrombolytic therapy (n = 32), and unavailability of troponin results (n = 8) were excluded. As a result, a total of 241 AIS patients who underwent intravenous thrombolysis were included. Figure S1 summarizes the screening flowchart of included participants.

Of the 241 patients analyzed, 65 had hs‐cTnT levels above the reference (>14 ng/L, 27.0%). Patients in hs‐cTnT elevated group were significantly older than the normal group (71.0 ± 10.7 vs 62.5 ± 9.5, *P* < .001), while sex was equally distributed in each group. The severity of stroke assessed with admission NIHSS score was significantly higher in hs‐cTnT elevated group (5.0 [IQR 2.0‐10.5] vs 3.0 [IQR 2.0‐6.0], *P* = .010). Patient comorbidities, such as diabetes mellitus (*P* = .015), CAD (*P* = .008), and AF (*P* = .002), as well as laboratory abnormalities, such as higher serum creatinine levels (*P* = .008) and lower eGFR (mL/min/1.73 m^2^; *P* < .001) were more frequent in the hs‐cTnT elevated group. Insular cortex lesion, but not specifically right insular involvement, was significantly associated with hs‐cTnT elevation (26.2% vs 11.9%, *P* = .007). TOAST etiology differed between hs‐cTnT normal and elevated patients (*P* = .001). Etiology was more often considered cardioembolic in the elevated hs‐cTnT patients and more often macroangiopathic in the normal hs‐cTnT patients. Table 1 summarizes the baseline characteristics of patients in each group.

**Table 1:** Clinical characteristics of patients with and without elevated hs‐cTnT

Variable	hs‐cTnT >14 ng/L (n = 65)	hs‐cTnT ≤14 ng/L (n = 176)	*P*‐value
Age, years, mean (SD)	71.0 (10.7)	62.5 (9.5)	<.001
Male sex, n (%)	49 (75.4)	130 (73.9)	.811
ODT, minutes, mean (SD)	118.7 (61.7)	109.1 (56.1)	.253
DNT, minutes, mean (SD)	66.9 (25.7)	65.0 (27.6)	.620
ONT, minutes, mean (SD)	185.7 (61.0)	174.7 (57.3)	.198
NIHSS on admission, median (IQR)	5.0 (2.0‐10.5)	3.0 (2.0‐6.0)	.010
History			
Smoking, n (%)	28 (43.1)	94 (53.4)	.155
Alcohol abuse, n (%)	20 (30.8)	62 (35.2)	.517
Hypertension, n (%)	48 (73.8)	115 (65.3)	.210
Hyperlipidemia, n (%)	33 (50.8)	87 (49.4)	.854
Diabetes mellitus, n (%)	25 (38.5)	40 (22.7)	.015
Coronary artery disease, n (%)	25 (38.5)	38 (21.6)	.008
Previous MI history, n (%)	7 (10.8)	9 (5.1)	.118
Atrial fibrillation, n (%)	16 (24.6)	16 (9.1)	.002
Congestive heart failure, n (%)	8 (12.3)	11 (6.3)	.121
Previous stroke, n (%)	17 (26.2)	44 (25.0)	.855
Chronic obstructive pulmonary disease, n (%)	0 (0.0)	2 (1.1)	.388
Epilepsy, n (%)	1 (1.5)	2 (1.1)	1.000
Creatinine, μmol/L, mean (SD)	81.2 (31.2)	71.9 (20.3)	.008
eGFR (mL/min/1.73 m^2^), mean (SD)	81.5 (22.1)	94.5 (19.7)	<.001
Glucose (mmol/L), mean (SD)	8.8 (3.7)	7.9 (3.3)	.092
Insular cortex involvement, n (%)	17 (26.2)	21 (11.9)	.007
Right insula involvement, n (%)	8 (47.1)	15 (71.4)	.127
Stroke etiology (TOAST)			.001
Cardioembolic, n (%)	36 (55.4)	72 (40.9)	
Small‐vessel disease, n (%)	11 (16.9)	9 (5.1)	
Large‐artery arteriosclerosis, n (%)	13 (20.0)	75 (42.6)	
Undefined, n (%)	5 (7.7)	18 (10.2)	
Other defined, n (%)	0 (0.0)	2 (1.1)	

Abbreviations: eGFR, estimated glomerular filtration rate; hs‐cTnT, high‐sensitivity cardiac troponin T; IQR, interquartile range; MI, myocardial infarction; NIHSS, National Institutes of Health Stroke Scale; ODT, onset‐to‐door time; DNT, door‐to‐needle time; ONT, onset‐to‐needle time.

### 3.2 END and hemorrhagic transformation in patients with and without hs‐cTnT elevation

There were three patients who (4.6%) demonstrated END in the elevated hs‐cTnT group compared with eight patients (4.5%) in the normal hs‐cTnT group, showing no statistical significance. Since hemorrhagic transformation (HT) is an important cause of END, we also assessed the occurrence of asymptomatic hemorrhage, symptomatic hemorrhage, HT types, and remote parenchymal hemorrhage. Similarly, we failed to observe statistical significance in any of these categories, despite the overall HT showing an increasing trend in hs‐cTnT elevated patients (13.8% vs 7.4%, *P* = .128, OR 2.016, 95% CI 0.817‐4.969, Table 2).

**Table 2:** Univariate analysis of END or HT in patients with elevated or normal hs‐cTnT levels

Variable	hs‐cTnT >14 ng/L (n = 65)	hs‐cTnT ≤14 ng/L (n = 176)	*P*‐value	OR (95% CI)
Early neurological deterioration, *n* (%)	3 (4.6)	8 (4.5)	.982	1.016 (0.261, 3.953)
Hemorrhagic transformation, *n* (%)	9 (13.8)	13 (7.4)	.128	2.015 (0.817, 4.969)
Symptomatic ICH, *n* (%)	3 (4.6)	5 (2.8)	.499	1.655 (0.384, 7.130)
Asymptomatic ICH, *n* (%)	6 (9.2)	8 (4.5)	.176	2.136 (0.711, 6.411)
Hemorrhagic transformation types				
HI‐1 (hemorrhagic infarction‐1), *n* (%)	1 (11.1)	2 (15.4)	.190	
HI‐2 (hemorrhagic infarction‐2), *n* (%)	1 (11.1)	3 (23.1)		
PH‐1 (parenchymal Hemorrhage‐1), *n* (%)	3 (33.3)	0		
PH‐2 (parenchymal Hemorrhage‐2), *n* (%)	4 (44.4)	8 (61.5)		
Remote parenchymal hemorrhage, *n* (%)	2 (3.1)	3 (1.7)	.513	1.831 (0.299, 11.21)

Abbreviations: END, early neurological deterioration; HT, hemorrhagic transformation; hs‐cTnT, high‐sensitivity cardiac troponin T; ICH, intracerebral hemorrhage.

### 3.3 Mortality and disability in patients with and without hs‐cTnT elevation

We also evaluated whether increased levels of hs‐cTnT were associated with death and disability. In the univariate model, elevated levels of hs‐cTnT were associated with death at 14 days (7.7% vs 1.1%, *P* = .020, OR 7.250, 95% CI 1.370‐38.36), 30 days (12.3% vs 1.7%, *P* = .003, OR 8.094, 95% CI 2.077‐31.54), and 90 days (13.8% vs 2.3%, *P* = .002, OR 6.911, 95% CI 2.049‐23.31) after thrombolysis. Major disability (mRS 3‐5) similarly increased in hs‐cTnT elevated patients at 14 days (43.1% vs 20.5%, *P* < .001, OR 2.943, 95% CI 1.595‐5.430), but not at 30 days (*P* = .134) or 90 days (*P* = .054). Conversely, patients with increased levels of hs‐cTnT demonstrated an increased occurrence of the composite unfavorable outcome (mRS 3‐6) at 14 days (50.8% vs 21.6%, *P* < .001, OR 3.745, 95% CI 2.046‐6.855), 30 days (40.0% vs 20.5%, *P* = .003, OR 2.593, 95% CI 1.399‐4.804), and 90 days (41.5% vs 18.8%, *P* < .001, OR 3.079, 95% CI 1.653‐5.734) (Table 3).

**Table 3:** Functional outcome of patients with and without elevated hs‐cTnT

Variable	hs‐cTnT >14 ng/L	hs‐cTnT ≤ 14 ng/L	Unadjusted	*P*‐value	Adjusted (model 1)	*P*‐value	Adjusted (model 2)	*P‐*value
	(n = 65)	(n = 176)	OR (95%CI)	Univ.	OR (95%CI)	Multiv.	OR (95%CI)	Multiv.
Death								
14 days	5 (7.7)	2 (1.1)	7.250 (1.370, 38.360)	.020	3.236 (0.507, 20.660)	.214		
30 days	8 (12.3)	3 (1.7)	8.094 (2.077, 31.540)	.003	4.545 (1.003, 20.600)	.049		
90 days	9 (13.8)	4 (2.3)	6.911 (2.049, 23.310)	.002	3.835 (1.003, 14.660)	.049		
Major disability (mRS 3‐5)								
14 days	28 (43.1)	36 (20.5)	2.943 (1.595, 5.430)	<.001	2.677 (1.177, 6.092)	.019	2.834 (1.227, 6.548)	.015
30 days	18 (27.7)	33 (18.8)	1.660 (0.856, 3.219)	.134	0.987 (0.395, 2.463)	.977	1.092 (0.422, 2.825)	.857
90 days	18 (27.7)	29 (16.5)	1.941 (0.990, 3.807)	.054	1.198 (0.478, 2.999)	.700	1.372 (0.521, 3.615)	.522
Composite unfavorable outcome (mRS 3‐6)								
14 days	33 (50.8)	38 (21.6)	3.745 (2.046, 6.855)	<.001	3.525 (1.465, 8.479)	.005	3.976 (1.599, 9.887)	.003
30 days	26 (40.0)	36 (20.5)	2.593 (1.399, 4.804)	.003	1.599 (0.611, 4.190)	.339	1.911 (0.682, 5.355)	.218
90 days	27 (41.5)	33 (18.8)	3.079 (1.653, 5.734)	<.001	2.041 (0.800, 5.208)	.136	2.618 (0.944, 7.262)	.065

*Note*: Death adjusted for age and NIHSS on admission.

Model 1 adjusted for age, gender, eGFR, insular cortex involvement, NIHSS on admission.

Model 2 adjusted for model 1 + history of atrial fibrillation, congestive heart failure, coronary artery disease.

In multivariate regression analysis, although we found that hs‐cTnT was independently associated with death at 30 days (*P* = .049, OR 4.545, 95% CI 1.003‐20.60) and 90 days (*P* = .049, OR 3.835, 95% CI 1.003‐14.66), major disability at 30 days (model 1: *P* = .977; model 2: *P* = .857), and 90 days (model 1: *P* = .700; model 2: *P* = .522) showed no difference between the two groups (Table 3). Similarly, in hs‐cTnT elevated patients, composite unfavorable outcome following an increase at 14 days (model 1: *P* = .005, OR 3.525, 95% CI 1.465‐8.479; model 2: *P* = .003, OR 3.976, 95% CI 1.599‐9.887), did not reach statistical significance at 90 days (Table 3). Disease‐specific mortality was summarized in Appendix S1. Kaplan‐Meier survival analysis lasting 90 days showed a significantly lower survival for patients with elevated hs‐cTnT compared to those with normal hs‐cTnT (Figure S2).

### 3.4 Factors associated with hs‐cTnT elevation in AIS patients

Multivariate regression analysis was conducted to determine variables in thrombolyzed AIS patients independently associated with hs‐cTnT elevation. Variables with a statistical significance of *P* < .1 in the univariate comparison were included. Older age (*P* < .001, OR 1.087, 95% CI 1.038‐1.137) was found to be significantly associated with hs‐cTnT elevation. We also found that stroke etiology, according to TOAST criteria, showed a statistical difference between the two groups, after multivariable regression analysis (*P* = .024, OR 0.641, 95% CI 0.435‐0.944), indicating that patients with elevated hs‐cTnT were more prone to present with cardioembolic etiology (Table 4).

**Table 4:** Multivariable regression analysis of variables associated with hs‐cTnT elevation

Variable	*P*‐value	OR (95%CI)
Age	<.001	1.087 (1.038, 1.137)
Coronary artery disease	.403	1.371 (0.655, 2.867)
Atrial fibrillation	.169	2.023 (0.741, 5.520)
Creatinine (μmol/L	.147	1.020 (0.993, 1.047)
eGFR (mL/min/1.73 m^2^)	.981	1.000 (0.967, 1.035)
Diabetes mellitus	.068	2.188 (0.945, 5.065)
Glucose (mmol/L)	.362	1.050 (0.945, 1.167)
NIHSS on admission	.515	1.019 (0.963, 1.079)
Insular cortex involvement	.454	1.438 (0.556, 3.718)
Stroke etiology (TOAST)	.024	0.641 (0.435, 0.944)

Abbreviations: eGFR, estimated glomerular filtration rate; NIHSS, National Institutes of Health Stroke Scale; TOAST, Trial of ORG 10172 in the acute stroke treatment.

### 3.5 Predictive models of serum hs‐cTnT for mortality at 30 and 90 days

**Figure 1:**
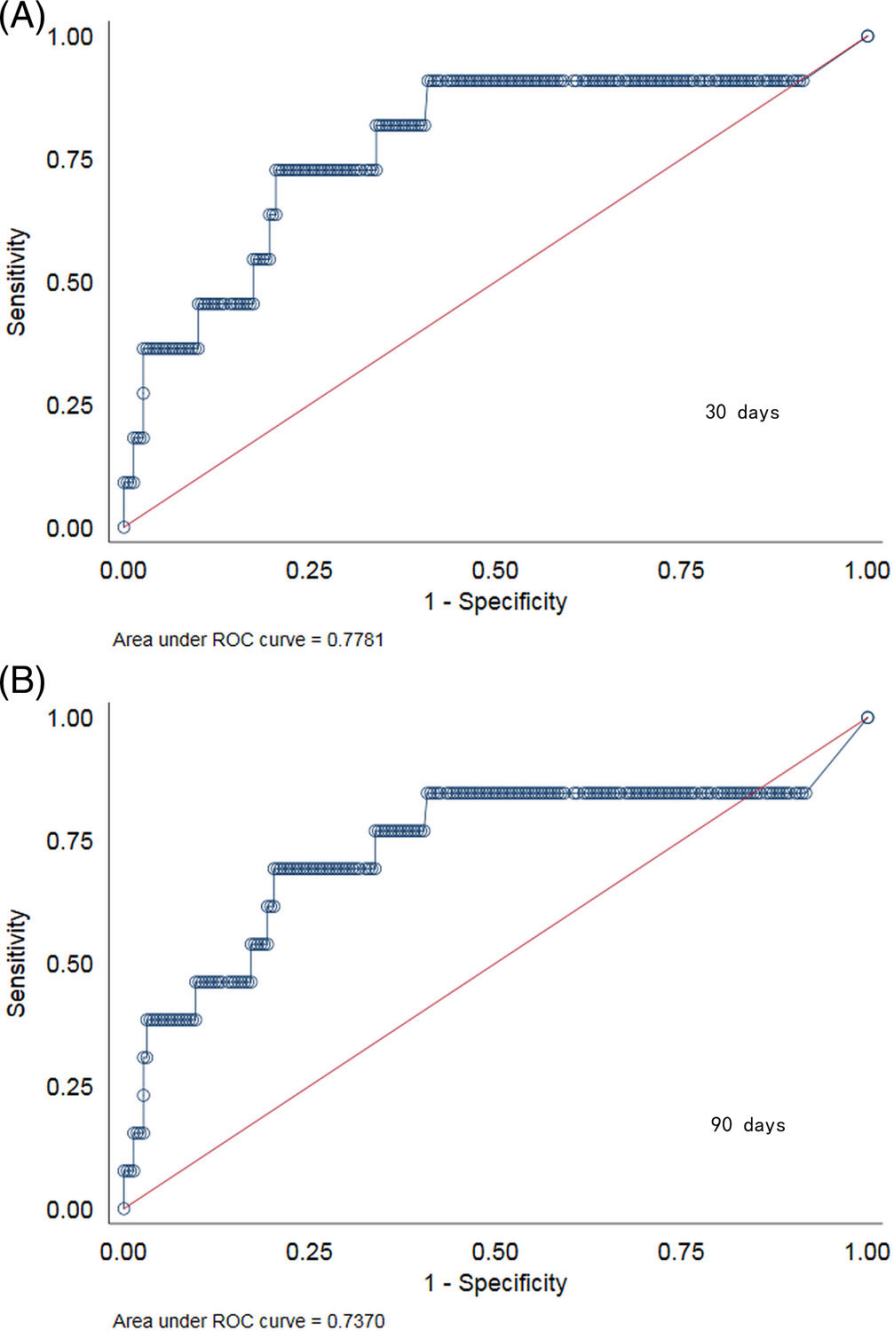
Receiver operating characteristics curves demonstrating the predictive value of high‐sensitivity cardiac troponin T at admission to predict 30‐ (A) and 90‐day (B) all‐cause mortality in acute ischemic stroke patients treated with thrombolysis

The observed area under curve (AUC) for 30 and 90 days mortality were 0.778 (95% CI 0.611‐0.945) and 0.737 (95% CI 0.554‐0.920), respectively. With Youden index of 0.523 for 30 days and 0.491 for 90 days, an identical optimal cutoff value of hs‐cTnT 15.39 ng/L (rounded up to 15.4 ng/L for clinical convenience) was determined, giving specificity of 0.796 and sensitivity of 0.727 for 30 days and specificity of 0.798 and sensitivity of 0.692 for 90 days (Figure 1).

## 4 DISCUSSION

In the present study, we found that in AIS patients who underwent intravenous thrombolysis, (a) elevation of serum hs‐cTnT was associated with 90‐day mortality after treatment, (b) elevation of hs‐cTnT was not associated with END or any type of HT, (c) older age and TOAST etiology (more cardioembolic etiology) were associated with hs‐cTnT elevation, and (d) hs‐cTnT ≥15.4 ng/L predicts mortality with acceptable sensitivities and specificities.

Stroke‐heart syndrome, in particular, ischemic stroke‐related cardiac alteration, may well be related to mechanisms of the brain‐heart connection (ie, the brain‐heart axis), which is the cortical modulation of the cardiovascular system.^2^, ^23^, ^24^ As a piece of rigorous evidence, damage to the insular cortex has been associated with myocardial injury, arrhythmia, and neuroendocrine disturbance.^11^, ^23^, ^25^ Despite being a potent candidate for diagnosing acute CAD, the diagnostic specificity of hs‐cTnT may be affected by many other disorders, such as respiratory or renal failure, ischemic or hemorrhagic stroke, and septic shock.^26^ Recent study even showed significant elevation of hs‐cTnT in healthy individuals with family history of heart disease.^27^ Specifically, the elevation of hs‐cTnT levels is common in AIS patients and may trigger exhaustive diagnostic investigations and consultations. In addition, therapies targeting ACS may be deleterious for AIS patients, due to the excessive intracranial hemorrhagic risk associated with anticoagulation, or the hypoperfusion risk with blood‐lowering therapy. Therefore, it is worthwhile to understand the implications and prognostic value of hs‐cTnT in AIS patients. To the best of our knowledge, this study is the first to evaluate the prognostic value of hs‐cTnT in AIS patients treated with IV tPA.

Prognosis of AIS is critical for not only patients and relatives but also physicians who have critical decision‐making power to optimize and allocate stroke care and resources.^28^ Instant biomarkers with desirable prognostic values can be of great help in distinguishing patients most likely to benefit from, or be harmed by, a particular therapy,^29^ including IV thrombolysis in AIS patients. In this regard, many blood biomarkers have been investigated, with proven potential in stroke diagnosis and recovery.^28^, ^30^, ^31^, ^32^ Multiple studies have demonstrated changes in a number of blood biomarkers, such as N‐terminal prohormone of brain natriuretic peptide (NT‐proBNP), copeptin, matrix metalloproteinase‐9 (MMP‐9), and S100 β, are all independently associated with various clinical outcomes.^28^ For instance, MMP‐9 is predictive of HT after IV thrombolysis, with higher MMP‐9 indicating more severe HT.^33^, ^34^ A very recent study showed an externally validated copeptin‐based risk score is strongly associated with several unfavorable outcomes, including death.^32^ Moreover, a panel of multiple biomarkers consisting of BNP, d‐dimers, MMP‐9, and S100 β protein was also shown to be valuable in detecting increased mortality after stroke.^31^ Unfortunately, to date, no blood biomarker in the field of stroke has been unequivocally considered as a validated surrogate marker, which can substitute for a clinical evaluation.^28^ Specifically, in this study, we found that a cutoff value of 15.4 ng/L for serum hs‐cTnT predicted both 30 and 90‐day mortality in AIS patients receiving IV thrombolysis. Because the level of hs‐cTnT can be affected by many comorbid conditions, we suggest the prediction model should be applied on an individual basis, weighing age, comorbidities, stroke severity, location of lesions, stroke etiology, and bleeding risk.

In patients without ACS, the prognostic performance of hs‐cTnT for all‐cause mortality seems superior to that of hs‐cTnI,^35^, ^36^ while the diagnostic value of cTnT and cTnI has been considered comparable in patients with ACS. In accordance with these findings, in AIS patients, the elevation of cTnT may be more sensitive to predict unfavorable outcomes as opposed to cTnI.^29^ A recent meta‐analysis showed that both cTnT and cTnI at baseline can independently predict an increased risk of all‐cause mortality in AIS patients, while efficacy of these predictors was not examined.^37^ Further studies need to be performed comparing these markers in AIS patients.

Serial measurement of cTn is the preferred examination of differentiating ACS and other confounding conditions.^38^ Dynamic changes of hs‐cTn levels in AIS patients are associated with unfavorable outcomes, including death.^6^, ^39^, ^40^ In line with previous studies of circulating cTnT, assessed with conventional or high‐sensitivity assay,^3^, ^5^, ^41^ we confirmed the elevation of serum hs‐cTnT is predictive of mortality in AIS patients. Unfortunately, patient information regarding the serial measurement of hs‐cTnT in this study was incomplete for statistical analysis.

Many factors influence the level of cTnT in patients with AIS. In our multivariable regression analysis, the independent association between older age and cTnT elevation in stroke patients is in line with previous studies.^42^, ^43^ We have also shown an independent association between cTnT elevation and stroke etiology, suggesting that etiology is more often considered cardioembolic in the elevated hs‐cTnT stroke patients. Previous studies also showed many causative factors related to hs‐cTnT elevation in AIS patients, such as insular cortex involvement, renal insufficiency, higher NIHSS score on admission, CAD, AF, and HF.^4^, ^10^, ^25^, ^44^ We included all the above variables to ensure the strength of the statistical analysis for unfavorable outcomes.

Some limitations of this study need to be mentioned. First, the recruited patients were mostly minor to mild stroke (refer to NIHSS at admission with IQR, Table 1). Therefore, the prognostic value of hs‐cTnT found in this study may not be applicable to patients with more severe ischemic stroke. Second, because the present cohort consisted of an ethnically homogeneous (100% Chinese) patient population, generalizability of the results was limited and results need to be validated in other populations. Third, no serial hs‐cTnT measurements were performed. Thus, we were unable to collect data on the dynamic pattern of hs‐cTnT, which might provide additional information about its prognostic performance. Lastly, because of relative small sample size of the study, covariates such as history of diabetes mellitus and stroke etiology were not adjusted in multiple regression models. Our future studies will focus on the prognostic value of cardiac troponins, including their dynamic changes, in AIS patients following endovascular therapies, which have become the standard of care for large vessel occlusions.

## 5 CONCLUSIONS

Elevation of hs‐cTnT occurs in more than one‐fourth of AIS patients treated with IV tPA and is independently associated with 3‐month mortality. Older age and cardioembolic etiology are independent determinants of hs‐cTnT elevation in these patients. Routine serum hs‐cTnT measurement in AIS patients who are indicated for IV thrombolysis may provide additional diagnostic and prognostic information. Furthermore, serum hs‐cTnT level > 15.4 ng/L may have a potential predictive value in risk stratification of AIS patients treated with IV tPA.

## CONFLICT OF INTEREST

The authors declare no potential conflict of interests.

## Supporting information


**FIGURE S1** Flow chart of the included study population

**FIGURE S2** Survival analysis comparing elevated vs normal high‐sensitivity cardiac troponin T (hs‐cTnT) group

**APPENDIX S1** Supplement with respect to methodology
