# Supplementary figures and images for: Elevation of high‐sensitivity cardiac troponin T at admission is associated with increased 3‐month mortality in acute ischemic stroke patients treated with thrombolysis
Source: Clin Cardiol. 2019 Jul 23;42(10):881–8. doi: 10.1002/clc.23237 (PMC6788486; doi:10.1002/clc.23237)

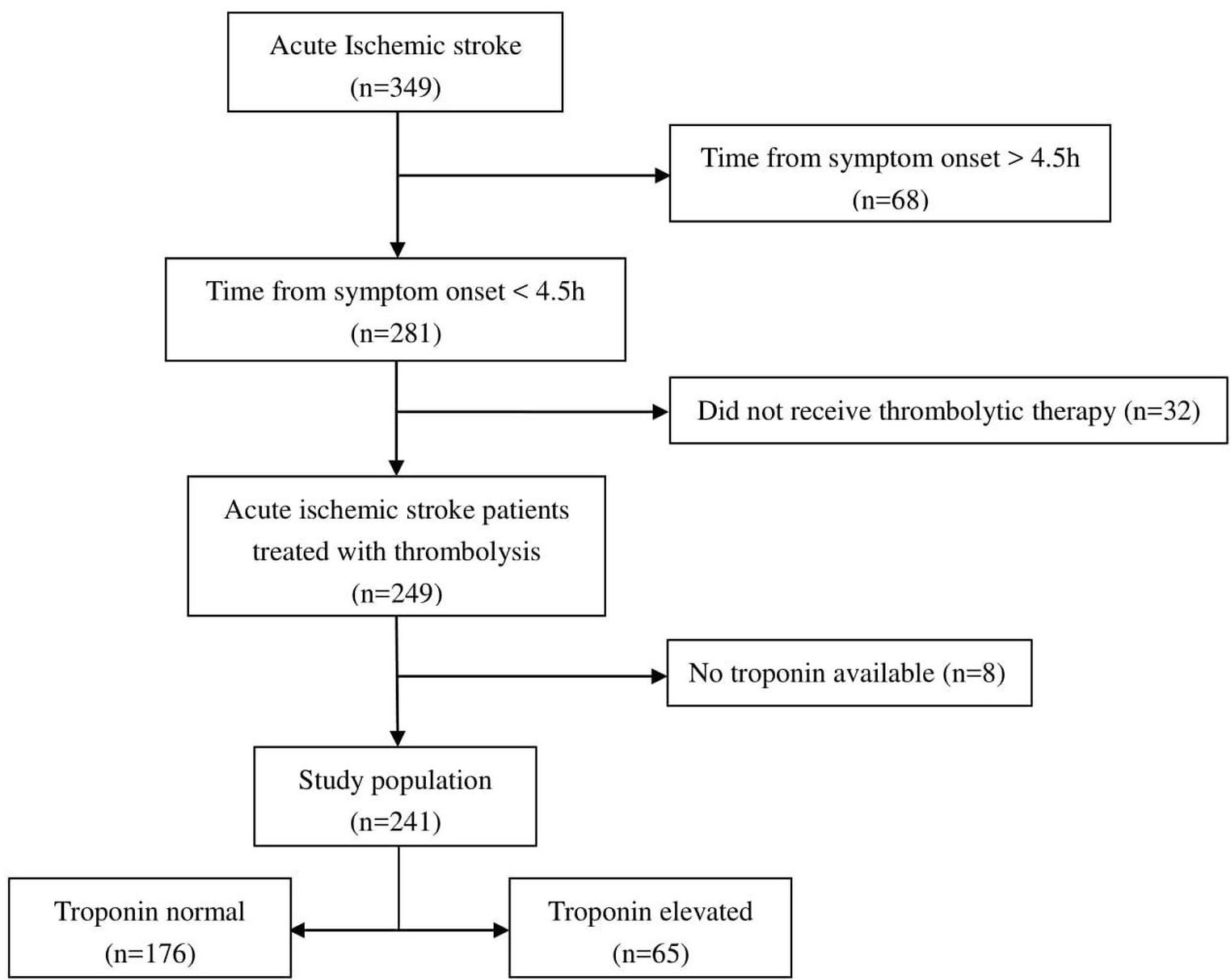

Supplement: Supplementary file 1 — FIGURE S1 Flow chart of the included study population [file CLC-42-881-s001.tif]

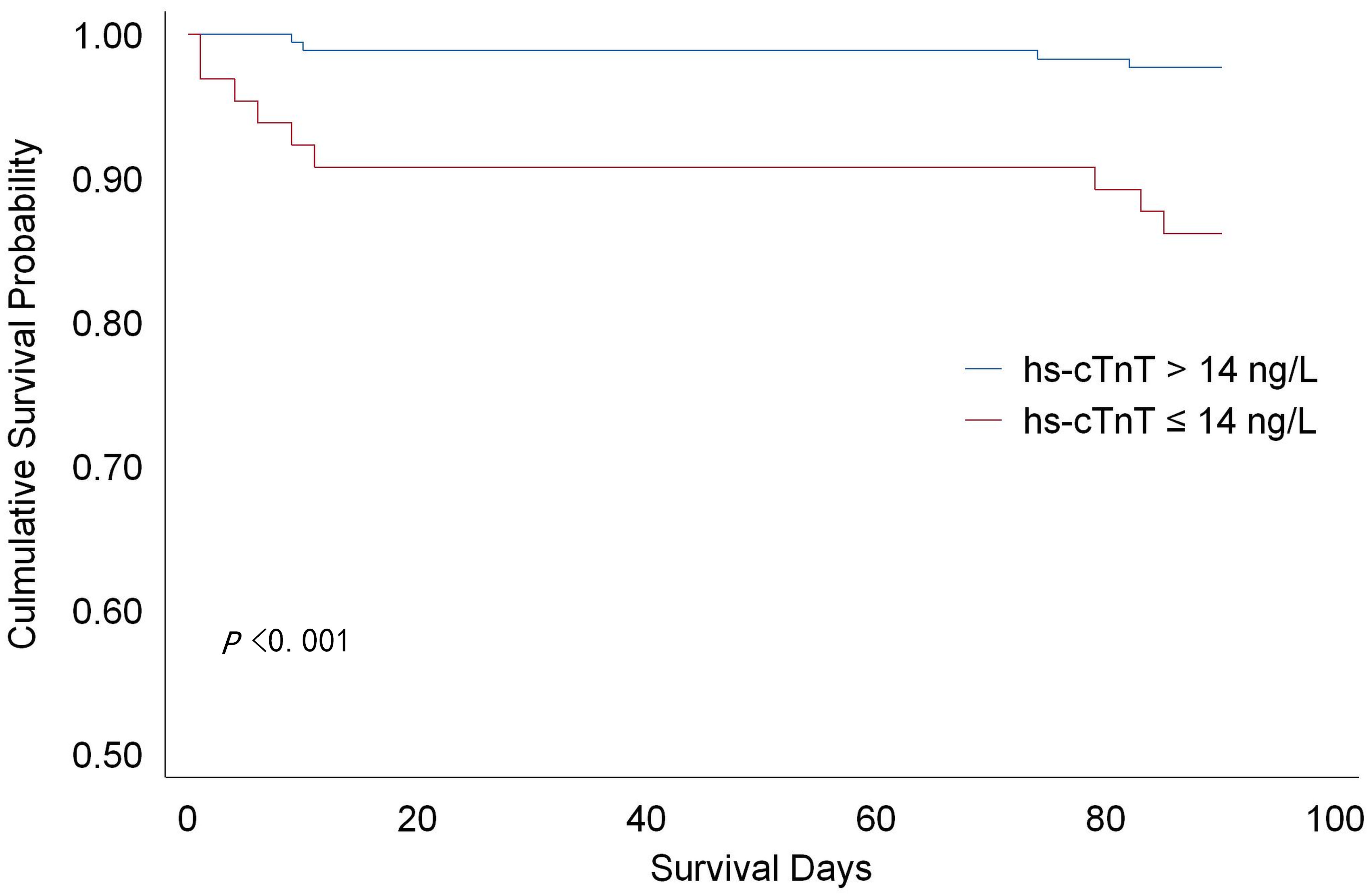

Supplement: Supplementary file 2 — FIGURE S2 Survival analysis comparing elevated vs normal high‐sensitivity cardiac troponin T (hs‐cTnT) group [file CLC-42-881-s002.tif]
