# Supplementary material for: Elevation of high‐sensitivity cardiac troponin T at admission is associated with increased 3‐month mortality in acute ischemic stroke patients treated with thrombolysis
Source: Clin Cardiol. 2019 Jul 23;42(10):881–8. doi: 10.1002/clc.23237 (PMC6788486; doi:10.1002/clc.23237)
Supplement: Supplementary file 3 — APPENDIX S1 Supplement with respect to methodology [file CLC-42-881-s003.docx]

**Supplement with respect to methodology**

The ECASS II criteria have been proven to be well correlated with a patient’s functional outcome at 90 days ([1](#_ENREF_1)) and demonstrates the highest inter-rater agreement ([2](#_ENREF_2)). Categories of intracranial hemorrhagic transformation (HT) were determined on the basis of non-contrast computed tomography (NCCT) scans, in accordance with the definition provided by ECASS trials ([3](#_ENREF_3), [4](#_ENREF_4)). Remote parenchymal hemorrhage (rPH) was defined as any intracranial parenchymal hemorrhagic lesion located remotely from infarcted brain tissue observed in the follow-up NCCT ([5](#_ENREF_5)).

Clinical outcomes and prognosis such as occurrence of END, sICH and death were all identified and assessed by stroke neurologists. Data was entered and managed by dedicated stroke neurologists using electronic database. Accuracy and completeness of variables were monitored and checked by dedicated follow-up nurses.

A computed tomography (CT) scan was repeated after 24−36 hours (or earlier in cases of rapid and/or severe clinical deterioration) and again between Day 4 and 7. All CT scans and neuroimaging assessment of insular cortical involvement were independently reviewed by one stroke neurologist and one neuroradiologist, who were both blinded to patient grouping.

Serum concentrations of hs-cTnT upon admission were measured using an Elecsys 2010 analyzer (cobas e 411; Elecsys, Roche Diagnostics, Mannheim, Germany). The blank limit was 3 ng/L (pg/mL). The upper reference limit (99^th^ percentile) was 14 ng/L, and the lowest concentration, with a coefficient of variation of 10%, was 13 ng/L.

Levels of serum creatinine were examined upon admission. The estimated glomerular filtration rate (eGFR) was calculated using the Chronic Kidney Disease Epidemiology Collaboration (CKD-EPI) formula. Standard 12-lead electrocardiograms (ECGs) were performed upon admission and interpreted by investigators blinded to patient data.

*Intravenous thrombolysis protocol*

After confirmation of thrombolysis eligibility ([6](#_ENREF_6)), AIS patients received intravenous tPA (0.9 mg/kg body weight) with 10% of the dosage as a bolus injection and 90% as an infusion over a period of 60 minutes. Post-IV tPA monitoring conformed to the recommendations of Early Management Guidelines of the AHA/ASA and lasted at least 24 hours ([6](#_ENREF_6)).

**Supplement addressing absence of analysis for disease-specific mortality**

Because only 13 patients (5.4%) died during 3 months follow up, disease-specific mortality was not analysed due to the limited death in each group. In elevated hs-cTnT group, nine death was respectively ascribed to cerebral infarction (6), sICH (2) and pneumonia (1), while in normal hs-cTnT group, four death was due to cerebral infarction (1), sICH (1), pneumonia (1) and multiple organ failure (1).

References

1. Rao NM, Levine SR, Gornbein JA, Saver JL: Defining clinically relevant cerebral hemorrhage after thrombolytic therapy for stroke: analysis of the National Institute of Neurological Disorders and Stroke tissue-type plasminogen activator trials. *Stroke*. 2014;**45**(9):2728-2733.

2. Yaghi S, Willey JZ, Cucchiara B, Goldstein JN, Gonzales NR*, et al.*: Treatment and Outcome of Hemorrhagic Transformation After Intravenous Alteplase in Acute Ischemic Stroke: A Scientific Statement for Healthcare Professionals From the American Heart Association/American Stroke Association. *Stroke*. 2017;**48**(12):e343-e361.

3. Hacke W, Kaste M, Bluhmki E, Brozman M, Davalos A*, et al.*: Thrombolysis with alteplase 3 to 4.5 hours after acute ischemic stroke. *N Engl J Med*. 2008;**359**(13):1317-1329.

4. Hacke W, Kaste M, Fieschi C, von Kummer R, Davalos A*, et al.*: Randomised double-blind placebo-controlled trial of thrombolytic therapy with intravenous alteplase in acute ischaemic stroke (ECASS II). Second European-Australasian Acute Stroke Study Investigators. *Lancet*. 1998;**352**(9136):1245-1251.

5. Trouillas P, von Kummer R: Classification and pathogenesis of cerebral hemorrhages after thrombolysis in ischemic stroke. *Stroke*. 2006;**37**(2):556-561.

6. Powers WJ, Rabinstein AA, Ackerson T, Adeoye OM, Bambakidis NC*, et al.*: 2018 Guidelines for the Early Management of Patients With Acute Ischemic Stroke: A Guideline for Healthcare Professionals From the American Heart Association/American Stroke Association. *Stroke*. 2018;**49**(3):e46-e110.
